# Towards a sweetpotato genomic-enabled breeding: optimizing two-stage analysis of multi-environment augmented trials

**DOI:** 10.1007/s00122-026-05204-x

**Published:** 2026-03-21

**Authors:** Saulo Chaves, Reuben Ssali, José Tiago B. Chagas, Kaio Olimpio G. Dias, Bert De Boeck, Thiago Mendes, Hannele Lindqvist-Kreuze, Hugo Campos, G. Craig Yencho, Guilherme da Silva Pereira

**Affiliations:** 1 Department of Agronomy, Federal University of Viçosa, Viçosa, MG Brazil; 2 International Potato Center (CIP), Kampala, Uganda; 3 Department of General Biology, Federal University of Viçosa, Viçosa, MG Brazil; 4 International Potato Center (CIP), Lima, Peru; 5 International Potato Center (CIP), Nairobi, Kenya; 6 Department of Horticultural Science, North Carolina State University, Raleigh, NC USA; 7 Department of Genetics, Luiz de Queiroz College of Agriculture, University of São Paulo, Piracicaba, SP Brazil

## Abstract

**Key message:**

Using the full weight matrix and deregressed pedigree-based best linear unbiased predictions in second-stage models lead to selections and genomic predictions closer to those obtained using a single-stage model.

**Abstract:**

In multi-environment genomic selection, although single-stage (SS) models are generally more efficient (no loss of information), there are contexts where they are difficult to fit, making two-stage models the most practical alternative. An example is the evaluation of early-stage observational trials (OTs) of sweetpotato breeding, where several clones are tested in unreplicated trials. In this study, 1,138 clones derived from partial diallels within two gene pools had their storage root yield evaluated across six OTs. Using this scenario, we compared the selection and prediction performances of models under different two-stage strategies against the SS benchmark. We also tested whether pool-specific genomic prediction models offered advantages over models trained with the complete dataset. Given the lack of replication in OTs, we hypothesized that deregressed best linear unbiased predictions (dBLUPs) or pedigree-based dBLUPs (dABLUPs) would work more appropriately as inputs for second-stage models than best linear unbiased estimates (BLUEs). These comparisons were conducted within weighted models using either a diagonal weight matrix or the full weight matrix. For selection, differences among second-stage models were minor, with a slight advantage for those using dABLUPs as entries, combined with the full weight matrix. For prediction, however, the choice of weighting scheme had a greater impact on performance than the choice of entry. Using the complete dataset, differences between entries were marginal, but for pool-specific predictions, dABLUPs provided the best performance. Overall, if adopting a two-stage strategy for the analysis of augmented trials, we recommend using dABLUPs together with the full weight matrix.

**Supplementary Information:**

The online version contains supplementary material available at 10.1007/s00122-026-05204-x.

## Introduction

Genomic selection (or selection based on genomic prediction) represented a paradigm shift in plant breeding. The possibility of selecting individuals based on their molecular marker data, rather than their phenotypic performance (Bernardo 1994; Meuwissen et al. 2001), opened the pathway for the so-called predictive breeding. Essentially, genomic selection acts as a surrogate of the phenotypic selection in specific breeding stages, decreasing the interval between generations and costs related to the management of field trials (Crossa et al. 2017). This is easier said than done, as several challenges must be overcome for efficient integration of genomic data into an existing breeding pipeline. To cite a few: how to deal with the genotype-by-environment interactions, since models trained in one environment may not be useful to others; and modelling strategies, i.e., selecting statistical methods that are accessible and can provide more complete and useful results in a timely manner.

There is a plethora of proposals for dealing with these barriers and integrating genomic selection into the breeding routines of the primary row crops, such as maize (Atanda et al. 2021), wheat (Juliana et al. 2020), and soybean (Wartha, Lorenz 2024). The decrease in costs of next-generation sequencing technologies has made it possible that genomic data are also available for orphan crops, like sweetpotato [*Ipomoea batatas* (L.) Lam., 2*n* = 6x = 90] (Zhang et al. 2024). Sweetpotato is one of the most important staple foods in developing countries, mainly in sub-Saharan Africa, where it is grown mostly by smallholder farmers (Low et al. 2017; Mwanga et al. 2017). It is relatively tolerant to extreme environmental conditions and highly nutritious, making it an important player in food security and sustainability (Kwak 2019). Efforts are therefore being made to cope with the typical obstacles to implement genomic selection despite sweetpotato’s genetic challenges (namely autopolyploidy, self-incompatibility and high heterozygosity), so that it can be properly incorporated into the species’ breeding pipelines. Ultimately, this will translate into a more efficient selection and a rapid deployment of improved varieties (Friedmann et al. 2018; Gemenet et al. 2020; Lindqvist-Kreuze et al. 2024).

The International Potato Centre (CIP) in Uganda has implemented a two-part strategy for its sweetpotato breeding program, namely population improvement and product development (Gaynor et al. 2017; Swanckaert et al. 2021). Each pipeline is composed of a series of trials where observational trials (OTs), preliminary yield trials (PYTs), and advanced yield trials (AYTs) are conducted with a decreasing number of clones, and increasing plot sizes, number of replicates and environments (Grüneberg et al. 2019). Typically, candidates that reach AYT from the population improvement pipeline can be selected to become parents for either population improvement or product development pipelines. As part of CIP’s accelerated breeding scheme, there is an effort towards selecting parents in earlier stages, such as PYT or even OT. Furthermore, all these trials are replicated across locations and seasons, rather than across years, decreasing generation intervals (Lindqvist-Kreuze et al. 2024). OTs usually consist of thousands of individuals. Due to logistical issues such as the availability of propagative material and space limitations, these candidates are tested as smaller, unreplicated plots in augmented design trials.

The OTs’ framework described above clearly demonstrate how genomic prediction can enhance the efficiency of the breeding process. For instance, one can obtain genomic estimated breeding values (GEBVs) from multi-environmental trials (METs) of unreplicated genotypes by better sampling the effects of alleles, which are replicated across individuals and environments. Genomic selection in METs can be performed using single-stage or two-stage approaches. Single-stage models are fitted by combining relationship matrices of both genotyped and ungenotyped samples (Aguilar et al. 2010; Christensen, Lund 2010), or even by disregarding the ungenotyped ones (Bančič et al. 2023; Tolhurst et al. 2019). These models are considered the gold-standard practices, as there is no loss of information during the estimation process since it accounts for the complete genetic variance–covariance structure (Fernández-González & Isidro y Sánchez, 2025; Gogel et al. 2018).

Despite the single-stage modelling superiority, there may be situations where breeders prefer using a two-stage strategy. Two-stage approaches obtain best linear unbiased estimates (BLUEs) or deregressed best linear unbiased predictions (dBLUPs) in the first stage and use them—often weighted by a precision metric—in a less computationally demanding second stage (Möhring, Piepho 2009; Smith et al. 2001a, b; Welham et al. 2010). The reasons for this involve (i) lack of computational resource, as single-stage models are much more demanding than second-stage models (Damesa et al. 2019; Verbyla 2023); (ii) data availability, for example, when analysing historical data that have only BLUPs or BLUEs available (Garrick et al. 2009; Krause et al. 2025; Piepho et al. 2014); and (iii) complexity of single-stage models, for instance, when there is a huge amount of phenotypic and genomic data, or several trials with different designs or particular spatial adjustments (Cullis et al. 1998).

Considering that the efficiency of the two-stage approach relies heavily on the quality of the adjusted means obtained in the first stage, our goal was to test the hypothesis that dBLUPs or pedigree-based dBLUPs (dABLUPs) would be more appropriate as inputs for second-stage models than BLUEs. These comparisons were conducted within weighted models using either a diagonal weight matrix or the full weight matrix and compared the outputs of these models against the ones of a single-step model, taken as benchmarks. It is important to note that OTs are traditionally designed for selecting test clones based on their total genotypic value, whereas parental selection, focused on additive value, typically occurs in later stages such as PYTs and AYTs. Recent efforts aim to shorten breeding cycles by enabling parental selection directly in OTs, leveraging genomic information and the inclusion of parent clones within these trials. This approach indirectly improves the estimation of additive effects, and the present study contributes to validating this strategy.

## Material and methods

### Plant material

**Fig. 1:**
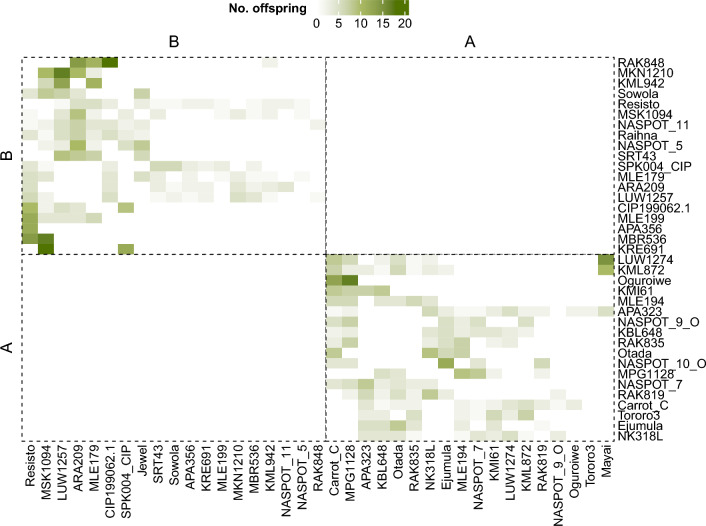
Heatmap representing the crossing scheme that originated the dataset. The right and lower borders have the parent names. The left and upper, as well as the inner dashed borders, distinguish the pools. The coloured cells represent the crosses, and the colour intensity, the number of offspring

The CIP-Uganda training population was composed of 1,138 sweetpotato genotypes, obtained from a partial diallel scheme within two newly proposed heterotic gene pools, named A and B with 19 and 20 parents, respectively. In total, there were 254 families, with number of offspring ranging from 1 to 18 (Fig. 1). These pools were established based on the multi-trait performance and estimated breeding values for priority traits, emphasizing yield and key biotic-stress targets relevant to Uganda (Swanckaert et al. 2024). They are a subset of the original gene pool described by David et al. (2018) using 31 simple sequence repeat markers. Therefore, the parents that composed the crossing blocks are putative holders of alleles that can increase storage root yield and/or sweetpotato virus disease (SPVD) resistance, in addition to having other commercially important traits, mainly related to storage root colour and shape (Mwanga et al. 2021). This population is part of the within-pool population development pipeline of a reciprocal recurrent selection scheme (Grüneberg et al. 2022).

### Phenotypic data

Six OTs were conducted over two years, 2021 and 2022, in three locations in Uganda: Namulonge—NAM (National Crops Research Institute—NaCRRI, at latitude 0.528916, longitude 32.612846, and altitude 1172 m above sea level, m.a.s.l), Serere—SER (National Semi-Arid Resources Research Institute—NaSARRI, at latitude 1.53735, longitude 33.44809, and altitude 1130 m.a.s.l.), and Rwebitaba—RWE (Zonal Agricultural Research and Development Institute, at latitude 0.693176, longitude 30.333027, and altitude 1506 m.a.s.l), over two seasons, first semester (OT1) or second semester (OT2). Hereinafter, we use the term “environment” as the season–location–year combination, such as OT1NAM21. The storage root yield was measured per plot and converted into tonnes per hectare (rytha) (Online Resource, Figure S1).

The OTs evaluated in this study were designed using the augmented row–column design proposed by Piepho, Williams (2016). Briefly, the design involves leveraging rows and columns in the randomization process to better sample field variation using the checks. In our case, ten contiguous rows constituted a row group, and ten contiguous columns constituted a column group. The interaction between row and column groups formed a block. The layout was randomized to ensure that each of the eight checks appeared once per block, three times per row group, and five times per column group, totalling 15 replicates per check. In addition to the checks, we evaluated 39 parents (Fig. 1), some of which were replicated within trials. Unlike the checks, the parents were not randomized according to the blocking structure; instead, they were assigned randomly across the field, similar to the unreplicated test clones. The number of replicates per parent ranged from 1 (unreplicated) to 15. Each observational trial (OT) comprised 50 rows and 30 columns, totalling 1,500 plots (Online Resource, Figure S2), with an average of 24% allocated to replicated treatments (checks or parents). The plots consisted of a single row with 10 plants, spaced by 0.3 m. The spacing between plots was of 1 m. The area of each plot was of 2.7 m^2^. The 1,138 test clones, 39 parents, and 8 checks were evaluated in all environments, representing a 100% connectivity. This percentage was not achieved after data collection due to missing data, mainly caused by mortality.

### Genomic data

Leaf samples of 1,125 test clones and 39 parents were collected and sent to DArT (Australia) for DNA extraction and genotyping using the DArTag panel, which has 3,120 SNPs (Zhao et al. 2024). The dosage data were processed and filtered out due to missing data (> 10%), monomorphism, and minor allele frequency (MAF < 0.05). At the end, 2,367 highly informative markers were selected to be used in the analyses described below.

### Statistical procedures

In the mathematical notation below, $M$ is the number of environments $\left(m=1, 2, \dots ,M\right),$ $V$ is the number of treatments (where ${v}_{p}=1, 2, \dots ,{V}_{p}=39$ parents and ${v}_{g}=1, 2,\dots ,{V}_{g}=\mathrm{1,138}$ test clones, totalling $v=1,2,\dots ,V={V}_{p}+{V}_{g}=\mathrm{1,177}$ treatments); $R$ is the number of rows, $C$ is the number of columns, and $N$ is the number of phenotypic observations, with $N=\sum_{m=1}^{M}{n}_{m}$, where ${n}_{m}$ is the number of plots per environment. All analyses described below were done using the R software environment, v. 4.4.1 (R Core Team 2024). The linear mixed models were solved using the algorithm implemented in ASReml-R v. 4.2 (The VSNi Team 2023), from which we obtained the variance component estimates via restricted maximum likelihood (REML, Patterson, Thompson 1971), and the best linear unbiased estimates (BLUEs) and predictions (BLUPs, Henderson 1975). The adjusted means to be used in the second stage (either BLUEs or deregressed BLUPs) were obtained using the package lmmtools (Verbyla 2025). All plots but the heatmaps [which were built using the package ComplexHeatmap (Gu 2022)] were made using the ggplot2 package (Wickham 2016), with add-ins of the packages ggpubr (Kassambara 2023), GGally (Schloerke et al. 2024), desplot (Wright 2023), ggh4x (Van de Brand 2025), and ggrepel (Slowikowski 2024).

#### Relationship matrices

Provided the pedigree and genomic information of the selection candidates, two relationship matrices were built, namely:

- *Numerator relationship matrix* (${\boldsymbol{A}}$): a matrix of order $V$ built from the pedigree (Online Resource, Figure S3). It has the *expected* additive relationship between genotypes, computed from the co-ancestry coefficient. Due to the polyploid nature of sweetpotato, ${\boldsymbol{A}}$ was built using the adjustments proposed by Kerr et al. (2012).
- *Genomic relationship matrix* (${\boldsymbol{G}}$): it contains the *realized* additive relationship between genotypes, estimated from molecular data (Online Resource, Figure S4). We used the method proposed by VanRaden (2008), with a slight adjustment to address sweetpotato’s polyploidy (Endelman et al. 2018):
  1 $$\boldsymbol{G} = \frac{\boldsymbol{WW}^{\prime }}{{\sum\nolimits_{u}^{U} 6 p_{u} \left( {1 - p} \right)_{u} }}$$

where $p$ is the major allele frequency, $U$ is the number of loci $\left(u=1, 2, \dots , U\right)$, and ${\boldsymbol{W}}$ is the centred matrix of markers. Thirteen test clones and the checks did not have molecular information, so ${\boldsymbol{G}}$ is a matrix of order $\breve{V} = 1164$, the number of genotyped individuals.

We built these matrices using the packages AGHmatrix (Amadeu et al. 2023) and ASRgenomics (Gezan et al. 2022).

#### First-stage analyses: single-environment models

In two-stage analysis, first-stage models are fitted separately for each environment. The main objective of these models is to obtain adjusted means and weights to be used in the second stage (Smith et al. 2001a, b). Even when using the single-stage strategy, performing single-environment analyses is useful for overviewing the data set and performing quality control. The baseline model for the single-environment analysis was the following:

2 $${\boldsymbol{y}} = \boldsymbol{1}\mu + {\boldsymbol{X}}_{1} {\boldsymbol{b}} + {\boldsymbol{X}}_{2} {\boldsymbol{t}} + {\boldsymbol{Z}}_{1} {\boldsymbol{g}} + {\boldsymbol{Z}}_{2} {\boldsymbol{r}} + {\boldsymbol{Z}}_{3} {\boldsymbol{c}} + {\boldsymbol{Z}}_{4} {\boldsymbol{rc}} + {\boldsymbol{\varepsilon}}$$

where ${\boldsymbol{y}}$ is the ${n}_{m}\times 1$ vector of phenotypic records, $\mu$ is the intercept, ${\boldsymbol{b}}$ is a $3\times 1$ vector of fixed effects of pool (A, B and C, a built-in “pool” for the checks), ${\boldsymbol{t}}$ is a $8\times 1$ vector of fixed effects of checks, ${\boldsymbol{g}}$ is a $V\times 1$ vector of genotypic (or genetic) effects, ${\boldsymbol{r}}$ and ${\boldsymbol{c}}$ are the $\frac{R}{10}\times 1$ and $\frac{C}{10}\times 1$ vectors of row group and column group random effects, respectively; ${\boldsymbol{r}}{\boldsymbol{c}}$ is the $\frac{R\times C}{100}\times 1$ vector of random block effects; and $\boldsymbol{\varepsilon}$ is the ${n}_{m}\times 1$ vector of random residuals. The capital letters ${\boldsymbol{X}}$ and ${\boldsymbol{Z}}$ denote the respective incidence matrices for fixed and random effects, with column counts matching the corresponding vectors and ${n}_{m}$ rows, and $\boldsymbol{1}$ is a ${n}_{m}\times 1$ vector of ones. We assumed that all random effects were outcomes of a zero-centred Gaussian distribution with variance ${\sigma }_{x}^{2}$, with $x$ representing the corresponding effect. The spatial trends were addressed in the residual part using a first-order autoregressive procedure, ${\boldsymbol{\varepsilon}}\sim \mathcal{N}\left\{\boldsymbol{0},{\sigma }_{\varepsilon }^{2}\left[{\boldsymbol{A}}{\boldsymbol{R}}1\left({\rho }_{r}\right)\otimes {\boldsymbol{A}}{\boldsymbol{R}}1\left({\rho }_{c}\right)\right]\right\}$, where ${\boldsymbol{A}}{\boldsymbol{R}}1\left({\rho }_{r}\right)$ and ${\boldsymbol{A}}{\boldsymbol{R}}1\left({\rho }_{c}\right)$ are the $R\times R$ and $C\times C$ autocorrelation matrices in the row and column directions, respectively (Gilmour et al. 1997). The autocorrelation coefficients were estimated using REML.

We fitted three different models based on Eq. 2. The main difference between them was how we treated the genetic effects. First, we fitted a model with fixed genotypic effects, from which we obtained BLUEs. Then, we treated ${\boldsymbol{g}}$ as random, with ${\boldsymbol{g}}\sim \mathcal{N}\left(\boldsymbol{0},{\sigma }_{g}^{2}{{\boldsymbol{I}}}_{V}\right)$ where ${\sigma }_{g}^{2}$ is the genotypic variance and ${{\boldsymbol{I}}}_{V}$ is an identity matrix of order $V$, and obtained BLUPs. Finally, we fitted a modified animal model partitioning the total genotypic effects into additive and non-additive effects (Oakey et al. 2006), i.e., ${{\boldsymbol{g}}}^{\prime}=\left({{\boldsymbol{a}}}^{\prime}, {{\boldsymbol{n}}{\boldsymbol{a}}}^{\prime}\right)$, ${{\boldsymbol{Z}}}_{1}=\left({{\boldsymbol{Z}}}_{{1}_{a}},\boldsymbol{ }{{\boldsymbol{Z}}}_{{1}_{na}}\right)$, and $$\mathrm{Var}\left({\boldsymbol{g}}\right)=\left[\begin{array}{cc}{\sigma }_{a}^{2}{\boldsymbol{A}}& \boldsymbol{0}\\ \boldsymbol{0}& {\sigma }_{na}^{2}{{\boldsymbol{I}}}_{V}\end{array}\right]$$, where ${\boldsymbol{a}}$ and ${\boldsymbol{n}}{\boldsymbol{a}}$ are $V\times 1$ vectors of additive and non-additive effects, respectively; ${{\boldsymbol{Z}}}_{{1}_{a}}$ and ${{\boldsymbol{Z}}}_{{1}_{na}}$ are ${n}_{m}\times V$ design matrices, which are similar to Equation’s 2 ${{\boldsymbol{Z}}}_{1}$; and ${\sigma }_{a}^{2}$ and ${\sigma }_{na}^{2}$ are the additive and non-additive genetic variances, respectively. For the two-stage framework, we were interested in the ABLUPs, i.e., the vector of additive effects $\left(\boldsymbol{a}\right)$.

In models where we treated ${\boldsymbol{g}}$ as random, we tested its significance using the likelihood ratio (LR) test. Since we were testing variance components which are bound to zero, the LR statistics followed a mixture of chi-square distributions, $\sum_{l}^{L}{2}^{-L}\left(\genfrac{}{}{0pt}{}{L}{l}\right){\chi }_{l}^{2}$, in which $L$ is the number of variance components under investigation (in our case, $l\in \left\{0,L=1\right\}$) (Self, Liang 1987; Stram, Lee 1994). We also computed the narrow-sense, ${h}^{2}$, and broad-sense heritabilities, ${H}^{2}$, as:

3 $$h^{2} = \frac{{{\Theta }\sigma_{a}^{2} }}{{{\Theta }\sigma_{a}^{2} + \sigma_{na}^{2} + \sigma_{\varepsilon }^{2} }} ;{\quad \quad}H^{2} = \frac{{\sigma_{g}^{2} }}{{\sigma_{g}^{2} + \sigma_{\varepsilon }^{2} }}$$

with $\Theta =\overline{D({\boldsymbol{A}})}-\overline{{\boldsymbol{A}} }$ being a correcting factor, with $D()$ representing a function that extracts the diagonal elements of the corresponding matrix, and the bar notation representing the calculation of the mean. This adjustment is necessary to ensure that both ${\sigma }_{g}^{2}$ and ${\sigma }_{a}^{2}$ refer to the same reference population (Legarra 2016).

#### Preparations for the second stage: deregression and weights

BLUPs and ABLUPs were deregressed to avoid double shrinkage, generating dBLUPs and dABLUPs. This was done as described in Smith et al. (2001a, b) and Holland, Piepho (2024), and implemented in the package lmmtools (Verbyla 2025). Briefly, we fitted the model of Eq. 2 and obtained the REML-estimates of variance components. Then, we fitted a second model considering the genetic effects as fixed but fixing the variance components of the non-genetic effects using their previously REML-estimated values. Verbyla (2023) details the implications of this procedure and the mathematical proof that doing this removes the shrinkage bias. Notably, even though we employ the term “deregression” in this paper, the procedure described is not the same as the one proposed by Garrick et al. (2009), which uses individual entries and reliabilities. In fact, the last step to obtain entries for the second-stage analysis was fitting a model with *fixed* genetic effects, but with variance components of non-genetic random effects manually set. Therefore, we will refer to the adjusted values to be used in the second-stage model (BLUEs, dBLUPs or dABLUPs) as “entries”, denoted as $${{\boldsymbol{y}}}^{\star}=\left[\begin{array}{cccc}{y}_{1}^{\star} & {y}_{2}^{\star} & \dots & {y}_{V}^{\star}\end{array}\right]^{\prime}$$.

The next step was to obtain the weights. To avoid information loss, it is recommended to carry the full variance–covariance matrix of entries ($\boldsymbol{\Omega }$) to the second stage (Gogel et al. 2018; Piepho et al. 2012). For a single environment, $\mathrm{Var}\left({{\boldsymbol{y}}}_{m}^{\star }\right)={\boldsymbol{\Omega }}_{m}$; and for all environments, ${{{\boldsymbol{y}}}^{\star }}^{\prime}=\left({{\boldsymbol{y}}}_{1}^{{\star }{\prime}},{{\boldsymbol{y}}}_{2}^{{\star }{\prime}},\dots ,{{\boldsymbol{y}}}_{m}^{{\star }{\prime}}\right)$ and $\mathrm{Var}\left({{\boldsymbol{y}}}^{\star }\right)=\boldsymbol{\Omega }={\oplus }_{m}^{M}{\boldsymbol{\Omega }}_{m}$. Note that $\boldsymbol{\Omega }$ is unknown and is usually replaced by a REML-estimated surrogate ${\boldsymbol{Q}}$ (adopting the notation of Buntaran et al. 2020). Another option is to take the diagonal elements of the inverse of ${\boldsymbol{Q}}$ as weights (Smith et al. 2001a, b), obtaining $D\left({{\boldsymbol{Q}}}^{-1}\right)$. This is done as a way of saving computational resources under the risk of penalizing the efficiency of the second-stage model. We compared second-stage models weighted by ${\boldsymbol{Q}}$ and $1/D\left({{\boldsymbol{Q}}}^{-1}\right)$.

#### Second-stage analyses: multi-environment model

Considering only genotypes that had genomic information, we used the entries obtained from the first stage and their respective weights to fit the following model:

4 $$\boldsymbol{y}^{\star } = \boldsymbol{1}\mu + \boldsymbol{Xe} + \boldsymbol{Zg} + \boldsymbol{s} + \boldsymbol{\varepsilon}$$

where ${{\boldsymbol{y}}}^{\star }$ is the ${\breve{V}} M \times 1$ vector of entries, ${\boldsymbol{e}}$ is the $M\times 1$ vector of fixed effects of environments, ${\boldsymbol{g}}$ is the $\breve{V} M \times 1$ vector of random genetic effects nested within environments, ${\boldsymbol{s}}$ is a $\breve{V} M \times 1$ vector that has the first-stage error (Endelman 2023; Fernández-González & Isidro y Sánchez, 2025), and $\boldsymbol{\varepsilon}$ is the $\breve{V} M \times 1$ vector of i.i.d. residuals. In this model, ${\boldsymbol{s}}$ has known distribution parameters, i.e., ${\boldsymbol{s}}\sim \mathcal{N}\left(\boldsymbol{0},{\boldsymbol{Q}}\right)$ or ${\boldsymbol{s}}\sim \mathcal{N}\left[\boldsymbol{0},1/D\left({{\boldsymbol{Q}}}^{-1}\right)\right]$. We partitioned ${\boldsymbol{g}}$ into additive and non-additive effects, as previously described. ${\boldsymbol{g}}$ is distributed as:

5 $${\boldsymbol{g}}\sim { \mathcal{N}}\left[ {\boldsymbol{0},\left( {\begin{array}{*{20}c} {{{\boldsymbol{\Sigma}}}_{{g}} \otimes {\boldsymbol{G}}} & \boldsymbol{0} \\ \boldsymbol{0} & {\sqrt {{\boldsymbol{D}}_{g} } \left[ {{\boldsymbol{I}}_{M} + { \varrho }\left( {{\boldsymbol{J}}_{M} - {\boldsymbol{I}}_{M} } \right)} \right]\sqrt {{\boldsymbol{D}}_{g} } \otimes {\boldsymbol{I}}_{{\breve{V} }} } \\ \end{array} } \right)} \right]$$

where ${\boldsymbol{\Sigma}_{g}}$ represents the factor-analytic covariance structure (Piepho 1997), with ${{\boldsymbol{\Sigma}}}_{{{g}}}=\boldsymbol{\Lambda }{\boldsymbol{\Lambda }}^{{\prime}}+\boldsymbol{\Psi }$, where $\boldsymbol{\Lambda }$ is a $M\times K$ vector of factor loadings $\left({\lambda }_{mk}\right)$, $K$ being the number of latent variables in a factor-analytic model $\left(k=1, 2,\dots K\right)$; and $\boldsymbol{\Psi }$ is a diagonal matrix of order $M$ with the lack of fit variances. The covariance structure adopted for the non-additive effects is the heterogeneous compound symmetry structure (Wolfinger 1996), with ${{\boldsymbol{D}}}_{g}$ representing a diagonal matrix of order $M$ with the per-environment non-additive genetic effects $\left({{\sigma}^{2}_{na_{m}}}\right)$; ${{\boldsymbol{J}}}_{M}$ is a square matrix of ones of order $M$, ${\boldsymbol{I}}$ is an identity matrix whose order is indicated by its subscript, $\varrho$ is a common genetic correlation between environments, and $\otimes$ is the Kronecker product. The option for this structure to model the non-additive effects was based on parsimony, since our main interest was in the additive effects.

An important decision in factor-analytic models is on how many latent variables to use. We made this selection based on the percentage of variance explained (%EV) by the latent variables, both in a per environment and overall context, respectively (Gogel et al. 2018):

6 $$\% EV_{m} = \frac{D\left( {\boldsymbol{\Lambda \Lambda }^{\prime } } \right)_{m}}{D\left( {\boldsymbol{\Sigma }_{g} } \right)_{m} } \times 100 ; {\quad} \% EV = \frac{1}{m}\sum\limits_{m = 1}^{M} \% EV_{m}$$

We selected the model that explained at least 90% of the total variance and had all environments with, at least, 60% of the variance explained. We also computed the Akaike information criterion (Akaike 1974) to compare the goodness-of-fit of models with different number of multiplicative terms.

Using the structured genetic variance–covariance matrix of the factor-analytic model $\left({\boldsymbol{\Sigma}}_{g}\right)$, we built a correlation matrix to investigate the genotype-by-environment interaction (GEI) in the dataset. This matrix was built as $\boldsymbol{\Delta }{{\boldsymbol{\Sigma}}}_{g}\boldsymbol{\Delta }$, with $\boldsymbol{\Delta }$ being a diagonal matrix whose elements are $\sqrt{D{\left({{\boldsymbol{\Sigma}}}_{g}\right)}^{-1}}$(Cullis et al. 2010). ${{\boldsymbol{\Sigma}}}_{g}$ was also used to calculate the measures of variance explained (main effects and GEI effects, and within it, the portion due to heterogeneity of scale and lack of genetic correlation) using the equations derived by Bančič et al. (2024) based on Cooper, DeLacy (1994). The main effects variance $\left({\sigma }_{G}^{2}\right)$ correspond to the mean of the upper triangular values of ${{\boldsymbol{\Sigma}}}_{g}$, which is equivalent to:

7 $$\sigma_{G}^{2} = \frac{{2\sum\nolimits_{{m < m^{\prime}}}^{M} {\sigma_{{g_{{mm^{\prime}}} }} } }}{M(M - 1)}$$

where ${{\sigma }_{g}}_{m{m}^{\prime}}$ is the covariance between environments $m$ and ${m}^{\prime}$. The GEI variance is obtained as:

8 $$\sigma_{GEI}^{2} = \frac{{\mathop \sum \nolimits_{m = 1}^{M} \sigma_{{g_{m} }}^{2} }}{M} - \sigma_{G}^{2}$$

where ${\sigma }_{{g}_{m}}^{2}$ is the genetic variance in environment $m$. Then, we can estimate the portion of ${\sigma }_{GEI}^{2}$ due to heterogeneity of scale as:

9 $$\sigma_{{GEI_{h} }}^{2} = \frac{{\sum\nolimits_{m = 1}^{M} {\left[ {\sigma_{{G_{m} }} - \left( {\frac{{\sum\nolimits_{m = 1}^{M} {\sigma_{{G_{m} }} } }}{M}} \right)} \right]^{2} } }}{M}$$

with ${{\sigma }_{G}}_{m}$ being the genetic standard deviation in environment $m$. The portion of ${\sigma }_{GEI}^{2}$ due to lack of genetic correlation is simply ${{\sigma }_{GEI_{l}}^{2}}={\sigma }_{GEI}^{2}-{{\sigma }_{GEI_{h}}^{2}}$. These values were computed using the R package FieldSimR (Werner et al. 2024).

#### Single-stage analysis: multi-environment model

Recent studies have shown that single-stage analyses are more efficient than two-stage approaches due to the loss of information inherent in the latter (Buntaran et al. 2020; Gogel et al. 2018; Verbyla 2023). Acknowledging this fact, we considered as “gold-standards” the results of the following single-stage model:

10 $${\boldsymbol{y}} = {\boldsymbol{Xf}} + {\boldsymbol{Z}}_{1} {\boldsymbol{g}} + {\boldsymbol{Z}}_{2} {\boldsymbol{r}} + {\boldsymbol{Z}}_{3} {\boldsymbol{c}} + {\boldsymbol{Z}}_{4} {\boldsymbol{rc}} + {\boldsymbol{\varepsilon}}$$

where ${\boldsymbol{y}}$ is the $N\times 1$ vector of phenotypic observations, ${\boldsymbol{f}}$ is the vector of combined fixed effects (intercept, pool, environment, checks, checks-by-environment interactions, and treatments without marker data), ${\boldsymbol{g}}$ is the $\breve{V} \times 1$ vector of genetic effects nested within environments, partitioned into additive and non-additive effects as previously described, and with distribution as in Eq. 5; ${\boldsymbol{r}}$, ${\boldsymbol{c}}$, and ${\boldsymbol{r}}{\boldsymbol{c}}$ are the design-related effects, all nested within environments, with zero-centred mean and block diagonal covariance structure [for example, ${\boldsymbol{r}}\sim \mathcal{M}\mathcal{V}\mathcal{N}\left( \boldsymbol{0}, {\oplus }_{m=1}^{M}{\sigma }_{r}^{2}{{\boldsymbol{I}}}_{m}\right)$]; and ${\boldsymbol{\varepsilon}}$ is the $N\times 1$ vector of residual effects, with ${\boldsymbol{\varepsilon}}\sim \mathcal{M}\mathcal{V}\mathcal{N}\left\{\boldsymbol{0},{\oplus }_{m=1}^{M}{\sigma }_{{\varepsilon }_{m}}^{2}\left[{\boldsymbol{A}}{\boldsymbol{R}}1{\left({\rho }_{r}\right)}_{m}\otimes {\boldsymbol{A}}{\boldsymbol{R}}1{\left({\rho }_{c}\right)}_{m}\right]\right\}$. The capital letters represent the incidence matrices. The purpose of using similar covariance structures for ${\boldsymbol{g}}$ in the single- and second-stage models is to make them as comparable as possible.

Using the single-stage model, we selected the number of multiplicative terms as previously described, using Eq. 6. Using the corresponding ${{\boldsymbol{\Sigma}}}_{{g}}$, we also built the correlation matrix and calculated the measures of variance explained (Eqs. 7, 8 and 9). Finally, we calculated the broad- and narrow-sense heritabilities as in Eq. 3, but with $\Theta =\overline{D({\boldsymbol{G}})}-\overline{{\boldsymbol{G}} }$.

#### Cross-validations

The predictive ability of the two-stage and single-stage models (Eqs. 4 and 10, respectively) was assessed using fivefold cross-validations (CV). This process is characterized by the random assignment of data points to folds of approximately equal size. At each round, one part (20% of the data points) had its values removed and predicted using the four other folds (80% of the data points) as a training set. All folds had the chance of acting as testing set, i.e., the whole dataset had predicted values after the end of five rounds.

When dealing with multi-environment trials, there are two possible scenarios: CV1 and CV2, adopting the nomenclature proposed by Burgueño et al. (2012). CV2 mimics a sparse-test setting, i.e., it predicts the performance of genotypes that were evaluated in some environments. In this case, the model leverages two pieces of information: the genetic covariances between relatives and the genotype’s performance per se in tested environments. CV1 simulates the prediction of new, untested genotypes. The sole source of information of CV1 is the genetic covariance between relatives (Burgueño et al. 2012).

Both cross-validations were repeated 30 times, with a different fold composition at each repetition. In the second-stage models, the predictive ability was assessed via Pearson’s correlation between observed and predicted values (instant correlation, as described in Zhout et al., 2017). The observed values in the second-stage models were the entries, BLUE, dBLUP, and dABLUP $\left({{\boldsymbol{y}}}^{\star }\right)$. In the single-stage framework, we used the individual theoretical accuracies as measures of predictive ability (Cappa et al. 2019; Putz et al. 2018), which is given by (adapted for hexapolyploidy):

11 $$r_{{ss_{v} }} = \sqrt {1 - \frac{{PEV_{v} }}{{\sigma_{a}^{2} \left( {1 + 5F_{v} } \right)}}}$$

where ${F}_{v}$ is the inbreeding coefficient. The term $(1+5{F}_{v})$ is obtained from the diagonal of ${\boldsymbol{G}}\boldsymbol{ }\left({G}_{vv}=\frac{\sum_{u}{\left({z}_{uv}-6{p}_{u}\right)}^{2}}{\sum_{{{u}}}6{p}_{u}(1-{p}_{u})}\right)$ (Endelman 2025)**.**

We also computed the mean-squared prediction error ($MSPE$), given by:

12 $$MSPE = \frac{1}{\breve{V}}\mathop \sum \limits_{{\breve{v} = 1}}^{{\breve{V}}} \left( {y_{v}^{{ \star }} - \widehat{a}_{v} } \right)^{2}$$

with ${\widehat{a}}_{v}$ being the GEBV of candidate $v$, and ${y}_{v}^{\star }$ the second-stage entry (BLUE, dBLUP, or dABLUP) or the corrected phenotype from single-stage models (i.e. ${y}_{v}^{\star }={y}_{v}-{f}_{v}$, with ${f}_{v}$ being the fixed effects of Eq. 10).

Given the structure of our population (two pools with weak relationship between them, as depicted in Online Resource Figures S3 and S4), we investigated if particularizing the second-stage genomic prediction models per pool would harm the predictive ability. In this case, we used the same model depicted in Eq. 4, but with dimensions corresponding to the number of genotypes from each pool. A positive result in this investigation (in comparison with using the complete dataset) would indicate that we could save computational resource without losing efficiency, at least prediction-wise.

## Results

### A brief note on model selection

The following subsections present results from the single-stage model (“SS”) and two-stage models (“2S”), the latter identified by the suffix “DW” or “FW” depending on whether a diagonal or full weight matrix was used. Comparisons are made against the SS model, which is widely regarded in recent literature as the gold standard for genetic evaluation in plant and animal breeding.

**Fig. 2:**
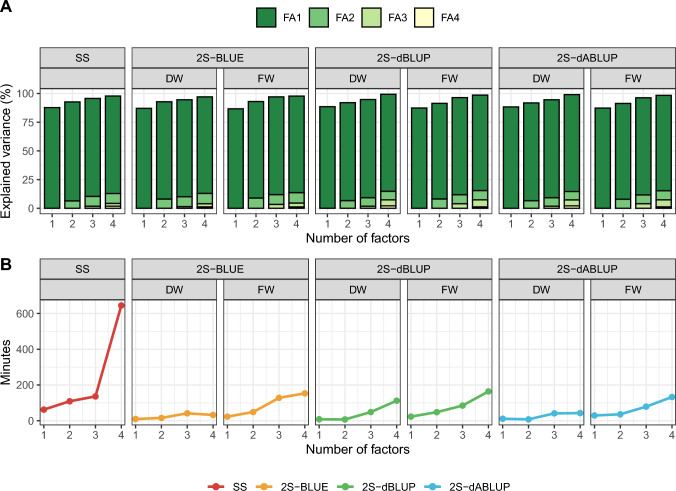
Single-stage (“SS”) and second-stage (prefix “2S”) model selection in the analysis of storage root yield data. **A** Percentage of explained variance (*y*-axis) per model with $K$ number of factors (*x*-axis) and per factor by model (colours inside the bars). **B** Elapsed time (in minutes, *y*-axis) per model with $K$ factors (*x*-axis). On the top of each cell, “DW” and “FW” refers to diagonal and full weights, used only in the second-stage models. “BLUE”, “dBLUP”, and “dABLUP” refer to the entry used in the second-stage models. In B, the elapsed time of “2S” models contains the summation of the time taken to fit all single-environment models and the second-stage model

The selected SS factor-analytic model, with two factors, explained 92.7% of the total variance (Fig. 2A) and had the lowest AIC among the tested models (Online Resource Table S1). Similarly, the chosen 2S factor-analytic DW and FW models also had two factors each, explaining between 91 and 95% of the total variance, respectively (Fig. 2A). Therefore, results shown below focus only on these selected models.

It is worth noting a practical motivation for using 2S analyses instead of SS models. Second-stage models required substantially less time and memory to run, even when summing the time taken to fit all single-environment models (about 4.4 min for BLUE models, 0.15 min for BLUP models, and 0.64 min for ABLUP models) and the second-stage model (Fig. 2B). Although advances in hardware and software are gradually reducing this limitation, computational efficiency remains important for democratizing genomic selection. For reference, all analyses were performed on a computer with 32 GB of RAM and a 12th Gen Intel Core i9-12900 K processor (base frequency 3.20 GHz), and runtime was monitored using the native *Sys.time()* function in R.

### Variance components and heritability

**Fig. 3:**
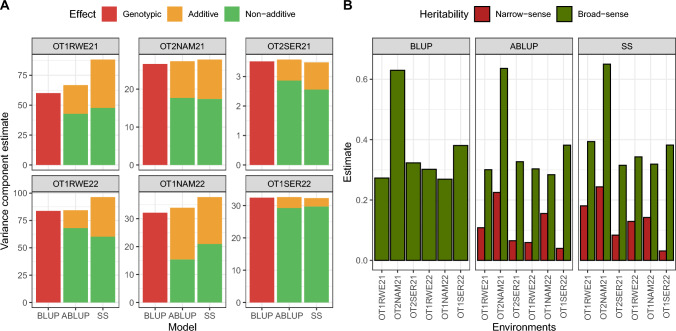
Variance components and heritabilities of first- and single-stage models in the analysis of storage root yield data. **A** Estimates of the genotypic, additive and non-additive genetic variance components. **B** Narrow- and broad-sense heritabilities. These estimates were obtained from a i) first-stage model that considered the genetic effect as random, but did not consider any relationship information, called BLUP model; ii) a first-stage modified animal model that used the relationship built from pedigree to model the genetic effects, called ABLUP model; and iii) a single-stage model, called SS, which used the genomic relationship matrix to model the genetic effects

All environments had significant genotypic, additive genetic and non-additive genetic effects (considering $\alpha =0.05$), which were somewhat equivalent $\left({\sigma }_{g}^{2}\approx {\sigma }_{a}^{2}+{\sigma }_{na}^{2}\right)$ to the first-stage models (BLUP and ABLUP in Fig. 3A). This equivalence is less clear considering the variance component estimates from the single-stage model (SS in Fig. 3A), mainly in trials conducted in Rwebitaba site (code RWE). It is worth mentioning that the single-stage model has a richer source of information to estimate variance components, namely the identity-by-state of candidates and data from multiple environments. Furthermore, the unreplicated setting of augmented designs may hinder variance component estimation, since replicated treatments (checks) are deemed as fixed and do not take part on the estimation procedure directly, although this effect may have been assuaged in this study due to replication of the clone parents. In other words, the estimates of the single-stage model tend to be less biased. The estimates of non-genetic variance component residual autocorrelations also varied per environment and model (Online Resource, Tables S2, S3 and S4).

The differences in the variance component estimates reflect on the narrow- and broad-sense heritabilities (Fig. 3B). Considering the BLUP model (first-stage, genotype effects as random but no relationship information), the broad-sense heritability ranged from 0.269 (OT1NAM22) to 0.630 (OT2NAM21). A similar trend was observed in the ABLUP model (0.284 to 0.636, same environments), while the SS model yielded higher estimates (0.315 of OT2SER21 to 0.650 of OT2NAM21). The influence of the non-additive effects on storage root yield was remarkable for most environments, as shown by the differences between the narrow- and broad-sense heritabilities, which ranged from 0.040 (OT1SER22) to 0.225 (OT2NAM21) in the ABLUP model, and from 0.031 to 0.244 in the SS model (same environments).

### Genotype-by-environment interaction

**Fig. 4:**
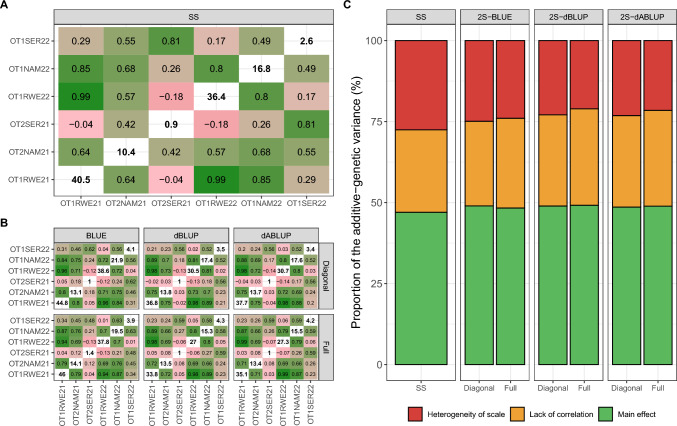
Genotype-by-environment interaction (GEI) investigation in the analyses of storage root yield data. **A** Additive genetic correlations between environments in the off-diagonal and additive genetic variances within environments in the diagonal, estimated using the single-stage model (SS). **B** Same but using second-stage models with different entries (BLUE, dBLUP, and dABLUP) and weight matrices (diagonal and full). **C** Partitioning of the additive genetic effects into its main andGEI counterparts. The GEI part is further subdivided into heterogeneity of scale and lack of correlation. The prefix “2S” in C refers to the two-stage strategy

Genotype-by-environment interaction (GEI) was not negligible in our dataset (Fig. 4), as shown by the range of additive genetic correlations from -0.04 to 0.99. Importantly, estimates from the single-stage model (Fig. 4A) and the second-stage models (Fig. 4B) followed very similar trends, regardless of the type of entry or whether a diagonal or full weight matrix was used. A few exceptions to this overall pattern were observed, such as the correlation between OT2NAM21 and OT2SER21, estimated at 0.42 in the single-stage model but ranging from 0.03 (2S-dBLUP-DW) to 0.12 (2S-BLUE-FW) in the second-stage models; and between OT2NAM21 and OT1SER22, estimated at 0.55 in the single-stage model and ranging from 0.23 to 0.45 in the second-stage models. Additive variance estimates (diagonal elements of Fig. 4A and B) showed clearer differences, both between the second-stage strategies and in comparison with the single-stage model.

Partitioning the additive genetic variance into main and GEI components revealed only subtle differences between single- and second-stage models (Fig. 4C). Approximately half of the additive variance was attributed to main effects (≈49%), which is favourable for selection, while the remaining ≈51% was due to GEI. This GEI portion was further divided (Eq. 9) into lack of genetic correlation (≈28%) and heterogeneity of scale (≈23%). The lack of genetic correlation represents the most problematic component, as it introduces the greatest uncertainty for both selection and prediction.

### Selections

**Fig. 5:**
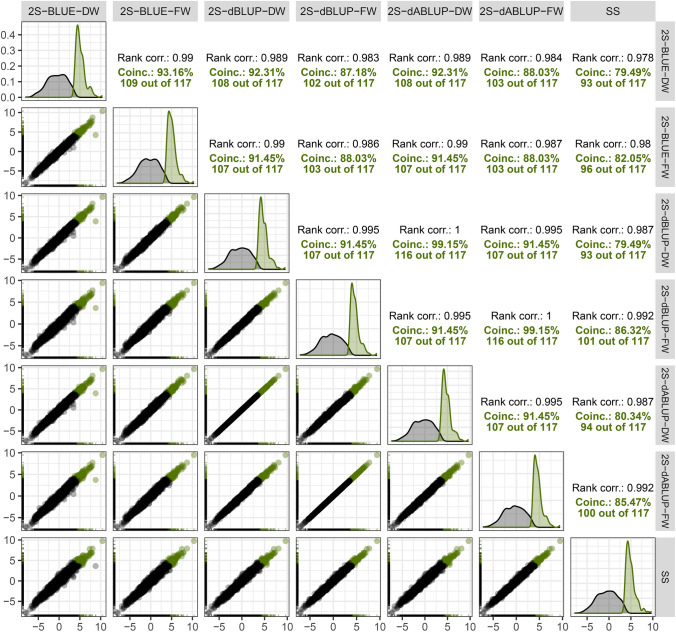
Relationship between genomic estimated breeding values (GEBVs) obtained from two- (prefix “2S”) and single-stage (“SS”) models. The column (top) and row (right) titles represent the adopted strategy. In two-stage strategies, “BLUE”, “dBLUP”, and “dABLUP” are the entries used in the second-stage model. “DW” and “FW” distinguish if a diagonal or a full weight matrix was used. The plots in the diagonal contain the distribution of all GEBVs (in black), and of the top 10% candidates (in green). The scatter plots in the lower triangle have the relationship between GEBVs of different models. Each grey dot represents a candidate’s GEBV, and the green dots are the candidates that were among the top 10% according to both strategies. This relationship is quantified in the upper triangle, which contains the ranking correlation (“Rank corr.”) between GEBVs (in black), and the coincidence between the top 10% candidates of both strategies

The high correlation between GEBVs from single- and second-stage models suggests that differences between them are negligible in terms of selection (Fig. 5). In practical terms, breeders would reach nearly the same conclusions regardless of the strategy adopted. However, when comparing coincidence in the top 10% selected individuals, differences became more apparent. In this case, the 2S-dBLUP-FW and 2S-dABLUP-FW strategies stood out, showing the closest agreement with the single-stage model. Another noteworthy point, further highlighted in the next subsection, is that second-stage models weighted with the full weight matrix consistently yielded better results, regardless of the entry type. A similar trend was observed when comparing the total genotypic value (TGV), which illustrates the selection for advancing breeding stages, which is normally performed at OTs (Online Resource, Figure S5). No strategy would provide selections 100% coincident, showcasing the sensibility of selection to the strategy adopted.

### Predictions

**Fig. 6:**
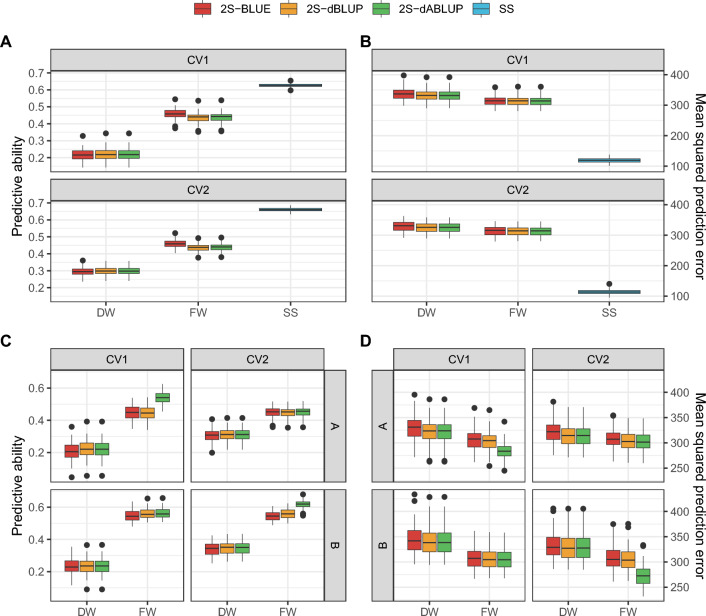
Cross-validation results of the across pool (**A** and **B**) and in the pool-specific (**C** and **D**) strategies: predictive ability (i.e. the correlation between the observed and predicted values, left *y*-axis) and mean-squared prediction error (MSPE, right *y*-axis) of single- (SS) second-stage models (prefix “2S”) that used different entries (BLUE, dBLUP, and dABLUP, boxes) and weight matrices (diagonal, DW, and full, FW, weight matrices, *x*-axis), in two cross-validation scenarios (CV1: prediction of new candidates; and CV2: sparse testing with 20% of missing data). These results refer to the storage root yield measured as tonnes per hectare

Proper weighting in second-stage models had a greater impact on prediction performance than the choice of entry (Fig. 6). Across all scenarios, FW models consistently showed higher predictive abilities and lower MSPE than DW models, regardless of entry. When comparing model types, SS models achieved the highest accuracy overall—evidenced by both higher predictive ability (Fig. 6A) and lower MSPE (Fig. 6B)—reinforcing their status as the gold-standard strategy. In CV1, among FW models, 2S-BLUE achieved the highest mean predictive ability, ~ 5% above the other strategies, while 2S-dABLUP produced the lowest mean MSPE, ~ 1% lower than the alternatives. Within DW models, 2S-dBLUP and 2S-dABLUP had comparable predictive ability, ~ 3% higher than 2S-BLUE, with 2S-dABLUP again achieving the lowest MSPE (2% and 1% lower than 2S-BLUE and 2S-dBLUP, respectively). In CV2, 2S-BLUE-FW outperformed 2S-dBLUP-FW and 2S-dABLUP-FW by ~ 5% in predictive ability, while 2S-dABLUP-FW maintained the lowest MSPE, ~ 1% lower than the others. Interestingly, DW models performed nearly identically in CV2, with a mean predictive ability of ~ 0.29 and mean MSPE of ~ 313.

Focusing on two-stage strategies, training models per pool seems to yield better results than using the complete information (Fig. 6C and D). This is probably due to the relatively poor relationship between candidates of different pools (Online Resource, Figures S3 and S4). Given this result, we recommend the usage of the pool-specific approach when adopting two-stage strategies, as it spends less computational resources and fits breeders’ expectations with the current pipeline. It is worth noting that, despite evaluating candidates from both pools together, the objective at this stage is to select within each pool.

The best-performing second-stage strategy for pool-specific predictions varied by pool and cross-validation scenario (Fig. 6B). With the full weight matrix, 2S-dABLUP often outperformed the other strategies. This advantage was evident in pool A under CV1, where 2S-dABLUP achieved mean predictive abilities 17% and 18% higher than 2S-BLUE and 2S-dBLUP, respectively, with nearly identical MSPE. A similar pattern was observed in pool B under CV2, where 2S-dABLUP surpassed 2S-BLUE and 2S-dBLUP, showing predictive abilities 11% and 9% higher, and MSPE values 11% and 10% lower, respectively. In the remaining scenarios, differences were more subtle, although a consistent trend emerged: 2S-dBLUP and 2S-dABLUP generally produced lower MSPE than 2S-BLUE.

## Discussion

### Two-stage strategies of multi-environment augmented trials

It is well-established in the literature that single-stage models provide the minimum loss of information, being the gold-standard procedure for analysing data from multi-environment trials (Gogel et al. 2018; Piepho et al. 2012; Smith et al. 2001a, b). The cross-validation results observed herein agree with this fact. Thus, why is fitting two-stage models is still an option? In observational trials of sweetpotato, as well as in early-stage trials of other crops, it is common to evaluate large numbers of candidates (> 1000) in non-orthogonal or unreplicated designs. Additionally, when working with historical data or data spanning different stages of a breeding programme, trials are often conducted under varying experimental designs. Although it is technically feasible to fit these data into single-stage models, doing so can be complex, computationally demanding (as shown in Fig. 2B), and challenging to parameterize effectively (Damesa et al. 2017; Fernández-González and Isidro y Sánchez, 2025; Verbyla 2023). In this context, it is important to evaluate how efficient simpler and more accessible approaches, such as two-stage analyses, can be, particularly since our overarching goal is to adapt the routine of a resource-limited breeding programme to incorporate genomic resources.

Stage-wise analysis is typically performed using BLUEs obtained from the first stage in the second-stage models. Nevertheless, in an unreplicated scenario, these BLUEs can be poorly estimated (Hoefler et al. 2020). In a way, unreplicated trials hold similarities to the animal breeding framework. Before making single-stage models with the relationship matrix encompassing both pedigree and marker information (the so-called ${\boldsymbol{H}}$ matrix) the standard procedure (Aguilar et al. 2010; Christensen, Lund 2010), animal breeders have used a two-stage strategy in which ABLUPs were obtained in the first stage, followed by their deregression prior to using them at the second stage (Aguilar et al. 2010; Misztal et al. 2020). The relationship information is a useful resource to address the lack of repetition of animals—which cannot be replicated—by leveraging the repetition of alleles, and the deregression avoids double shrinkage. Literature in deregression is abundant in animal breeding, and several alternatives to the “traditional” deregression (BLUP divided by its reliability, VanRaden 2001) have been proposed, with most of them being based on individual values (Calus et al. 2016; Garrick et al. 2009; Oliveira et al. 2018). Here, we use the procedure proposed by Smith Cullis, and Gilmour (2001), which completely removes the bias due to shrinkage, as proved by Verbyla (2023). Furthermore, we did not use the ${\boldsymbol{H}}$ matrix. Rather, we performed a single-stage GBLUP by moving the (few) ungenotyped individuals to the fixed part of the model (Tolhurst et al. 2019).

For selection purposes, 2S-dBLUPs and 2S-dABLUPs had closer results to those of SS (Fig. 5), mainly when the full weight matrix is used. Previous studies that observed the same trend pointed two main factors. First, the variance components of non-genetic effects in the first-stage models are better estimated when the genetic effect is considered random (Buntaran et al. 2020; Verbyla 2023). A model with better estimated variance components provides more accurate means to be used in the second stage. Second, there is limited loss of information when the complete variance–covariance matrix is taken from the first to the second stage (Damesa et al. 2017, 2019; Gogel et al. 2018). In this case, the second-stage model is deemed as “fully efficient”, since it is able to use a complete set of information (Damesa et al. 2017; Endelman 2023). In practical terms, selection based on a “fully efficient” two-stage strategy will not harm the breeder’s decision.

The second point cited in the previous paragraph was more evident in the cross-validation results (Fig. 6). Interestingly, the advantage of using full weights over diagonal weights was more evident here than in the work of Damesa et al. (2019), who also compared genomic predictions between these two types of models. This is probably due to differences in the experimental design, as they analysed data from trials laid out in alpha lattice, and we are dealing with augmented trials. In our case, the lack of replication and the missing-at-random pattern increased the importance of covariances between entries, available for the second stage only if the full weight matrix is used. Indeed, Fernández-González and Isidro y Sánchez (2025) showed in a simulation that differences between using diagonal weights and full weights are negligible when using orthogonal designs like RCBD. They also partitioned the genetic values into additive and non-additive effects, as performed here. This reinforces that the need of using full weights in second-stage models is driven by the unreplicated/augmented nature of our trials.

### Genotype-by-environment interaction in sweetpotato’s storage root yield

Genotype-by-environment interaction (GEI) is a key factor in plant breeding decision-making. Given the polygenic nature of storage root yield, it is not surprising that a large portion of its additive genetic variation is influenced by GEI, as previously reported (Mugisa et al. 2022; Swanckaert et al. 2020, 2025). In this study, the additive genetic correlation heatmaps (Fig. 4A and B) clearly distinguishes that environments that were in the same location had comparable performances. This is likely due to the distinct environmental conditions across sites: Namulonge has a humid climate and is more prone to sweetpotato virus disease (SPVD); Serere is drier and subject to drought stress, which can reduce performance unless genotypes possess tolerance; Rwebitaba is a highland (1,531 m.a.s.l) sweetpotato production system usually having longer physiological maturity period (7–8 months) due to cooler night temperatures (less than 12.7 ºC) and more prone to foliar Alternaria blight disease. Due to their different characteristics, each location requires a particular production system, adding a further confounding factor into GEI.

The predominance of genotype-by-location interactions underpins the “accelerated breeding scheme” (Grüneberg et al., 2019), which emphasizes multi-location over multi-year testing when advancing genotypes through breeding stages (Lindqvist-Kreuze et al. 2024). This strategy facilitates the early identification of stable clones. In contrast, if selection were based on a single location, such clones might be discarded, since stability and performance are not always strongly correlated. To effectively identify both high-yielding and stable clones, breeders must use tools that explicitly account for GEI. Factor-analytic linear mixed models provide valuable insights into GEI patterns, enabling the identification of genotypes adapted to specific environments or, more importantly, stable across the entire target population of environments (Meyer 2009; Smith et al. 2021; Smith, Cullis 2018). Tools that enrich FA models with additional information such as genomics and environmental data can enhance decision-making, ultimately improving the efficiency of the breeding process (Araújo et al. 2024; Mumford et al. 2023; Tolhurst et al. 2019, 2022).

### Towards a genomic-enabled sweetpotato breeding

With the molecular resources currently available for sweetpotato, the species has firmly entered the genomic era (for an overview, see Yencho et al. 2025). In this research, we showed a range of possible predictive abilities and MSPEs for storage root yield, depending on the scenario and the modelling strategy. A clear pattern is that sparse testing (CV2) is an easier scenario compared to the prediction of untested genotypes (CV1), as illustrated by the higher predictive abilities and lower MSPE of the latter compared to the former. These values and information are important as benchmarks for decision-making related to where (and how) genomic selection can be applied in sweetpotato’s breeding pipeline. This is important as there is an effort towards making genomic selection more common in the species’ breeding pipelines.

It is worth noting that this research is the first that used a proper breeding population tested in multiple environments to train genomic prediction models for storage root yield, and the values found here are probably closer to those found by a sweetpotato breeder when using a population with similar structure. It also validates the usage of the DArTag panel—a mid-density low-cost genotyping platform—for breeding purposes. The two previous study with genomic selection with sweetpotato used populations from different contexts. Gemenet et al. (2020) used a biparental population (Beauregard x Tanzania) and other genotyping platforms (DArTSeq and GBSPoly). They found predictive abilities for storage root yield lower to those observed in the present study, comparable to those obtained when diagonal weights were used. Batista et al. (2022) used a germplasm collection and analysed storage root colours, a highly heritable trait.

Many breeding programmes have yet to integrate genomic selection as a routine activity, as adapting an established pipeline is often challenging. This challenge is not unique to sweetpotato as it applies to other crops as well. Surely, dealing with real data complexity and taking the maximum amount of information from outputs are among the main challenges to this end. Thus, expanding this investigation to other economically important traits is an important part of the next steps of this study, which would also address the multi-trait nature of variety release. The final goal is to make genomic resources readily available for sweetpotato breeders in a simple yet efficient way, so breeding programmes can gradually transition from traditional to predictive breeding.

## Concluding remarks

In this study, and using storage root yield as an example, we showed how different two-stage genomic prediction strategies perform when dealing with unreplicated trials with complex designs. Trials of this kind are common not only in sweetpotato, but also in other clonally propagated species, like potato and sugarcane. Our results showed that the most important difference between second-stage modelling strategies is in the weighing: models with the full weight matrix provide results much more accurate than models with the diagonal weight matrix, regardless of the entry used in the second stage. The evident contrast exhibited herein is probably related to the unreplicated/augmented nature of our trials, given that other studies that performed a similar comparison in replicated/orthogonal designs reported more subtle differences. Regarding the entry type, there is gain—although marginal—when using deregressed BLUPs (pedigree-based or not), either for selection purposes, or in genomic prediction models, mainly in pool-specific predictions.

## Supplementary Information

Below is the link to the electronic supplementary material.

Supplementary file1 (PDF 12458 KB)

## Data Availability

The datasets generated and analysed during the current study, alongside the R files with the codes used for the analyses, are available in the GitHub repository https://github.com/ufv-molecular-breeding-lab/schaves_sweetpotato_two_stage.
