# Supplementary material for: Towards a sweetpotato genomic-enabled breeding: optimizing two-stage analysis of multi-environment augmented trials
Source: Theor Appl Genet. 2026 Mar 21;139(4):102. doi: 10.1007/s00122-026-05204-x (PMC13005861; doi:10.1007/s00122-026-05204-x)
Supplement: Supplementary file 1 — Supplementary file1 (PDF 12458 KB) [file 122_2026_5204_MOESM1_ESM.pdf]

Towards a sweetpotato genomic-enabled breeding: optimizing two-stage analysis of multi-environment augmented trials

Online Resource

Saulo Chaves Reuben Ssali José Tiago B. Chagas Kaio Olimpio G. Dias
Bert De Boeck Thiago Mendes Hannele Lindqvist-Kreuze Hugo Campos
G. Craig Yencho Guilherme da Silva Pereira

Abstract

In multi-environment genomic selection, although single-stage (SS) models are generally more efficient (no loss of information), there are contexts where they are difficult to fit, making two-stage models the most practical alternative. An example is the evaluation of early-stage observational trials (OTs) of sweetpotato breeding, where several clones are tested in unreplicated trials. In this study, 1,138 clones derived from partial diallels within two gene pools had their storage root yield evaluated across six OTs. Using this scenario, we compared the selection and prediction performances of models under different two-stage strategies against the SS benchmark. We also tested whether pool-specific genomic prediction models offered advantages over models trained with the complete dataset. Given the lack of replication in OTs, we hypothesized that deregressed best linear unbiased predictions (dBLUPs) or pedigree-based dBLUPs (dABLUPs) would work more appropriately as inputs for second-stage models than best linear unbiased estimates (BLUEs). These comparisons were conducted within weighted models using either a diagonal weight matrix or the full weight matrix. For selection, differences among second-stage models were minor, with a slight advantage for those using dABLUPs as entries, combined with the full weight matrix. For prediction, however, the choice of weighting scheme had a greater impact on performance than the choice of entry. Using the complete dataset, differences between entries were marginal, but for pool-specific predictions, dABLUPs provided the best performance. Overall, if adopting a two-stage strategy for the analysis of augmented trials, we recommend using dABLUPs together with the full weight matrix.

Summary

Overview of phenotypic data **2**

Field designs **3**

Population structure **4**

Factor analytic model selection **6**

Variance components of the first-stage analysis **7**

Model comparison: total genotypic value **13**

Overview of phenotypic data

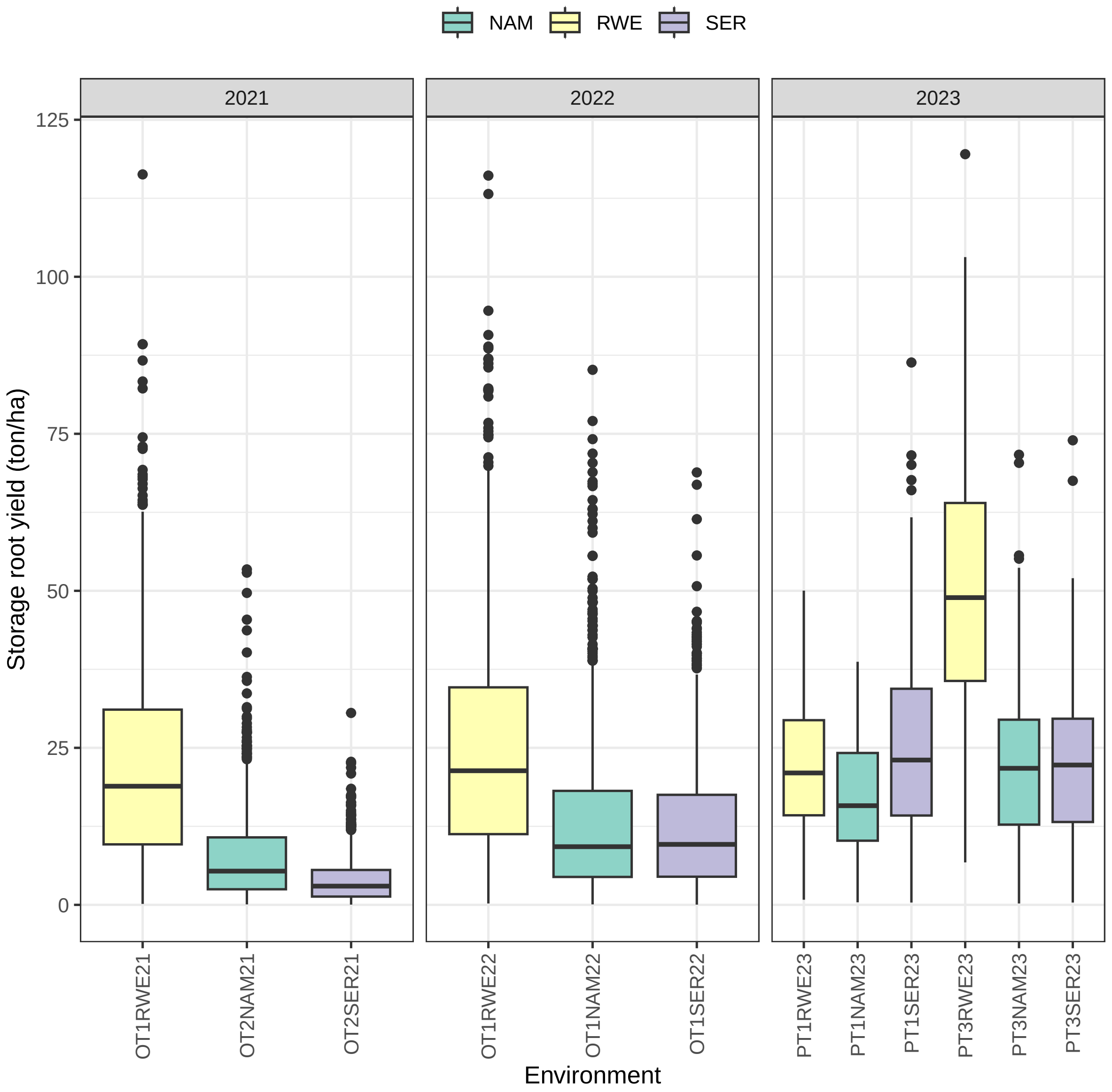

Figure S1: Box plots describing the raw phenotypic data (storage root yield in tons per hectare, y-axis) of each environment (x-axis). In the x-axis, the prefix 'OT' refers to observation trials (augmented desing), and 'PT', preliminary yield trials (p-rep design). The boxes are coloured according to the location: NAM (Namulonge), SER (Serere), RWE (Rwebitaba). The facets distinguish the year the trials was implemented.

Field designs

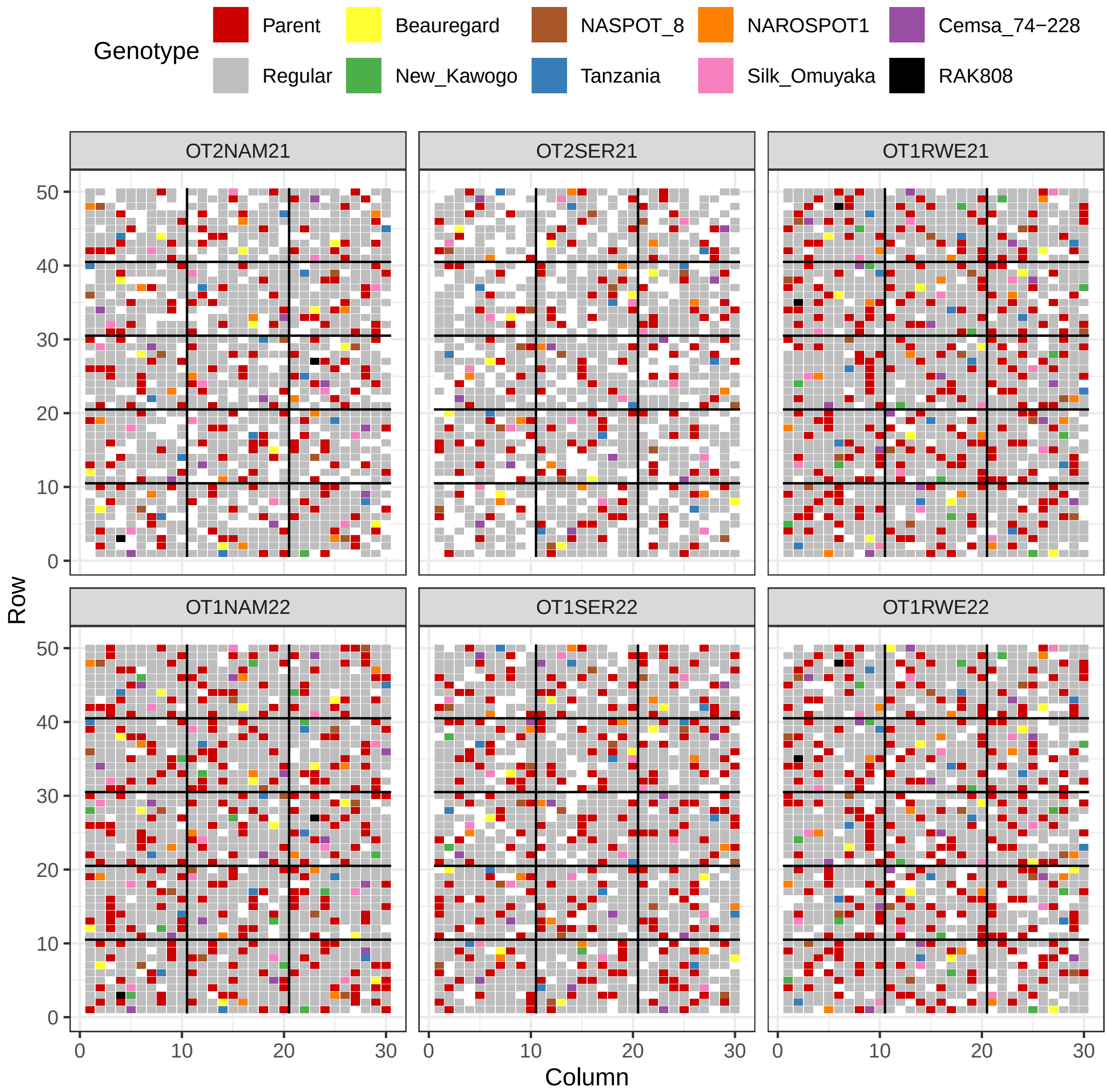

Figure S2: Representation of observational trials, implemented in augmented row-column design.

Population structure

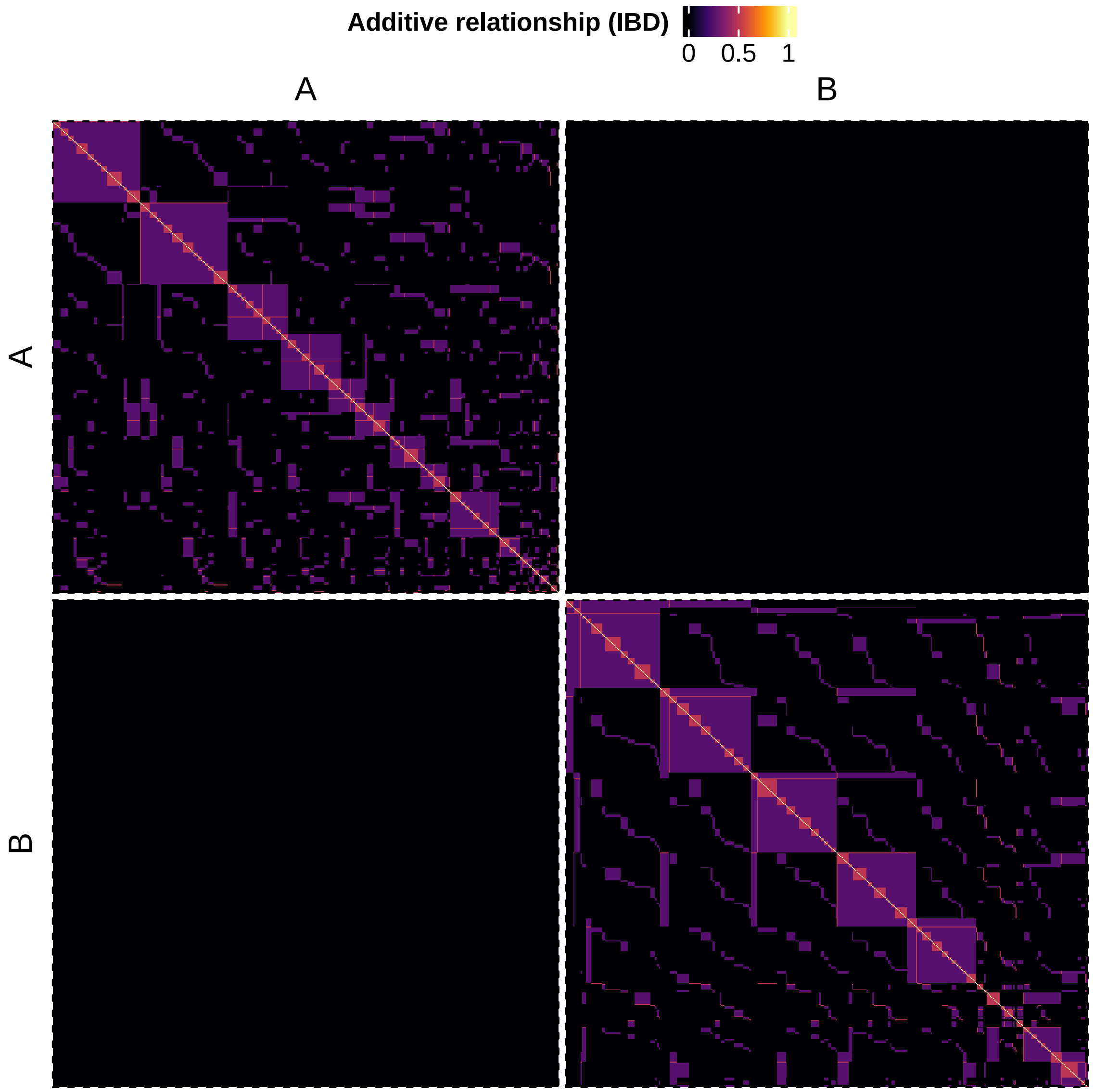

Figure S3: Heatmap representation of the numerator relationship matrix (identity-by-descent, IBD), subdivided according to the genetic pools (A and B)

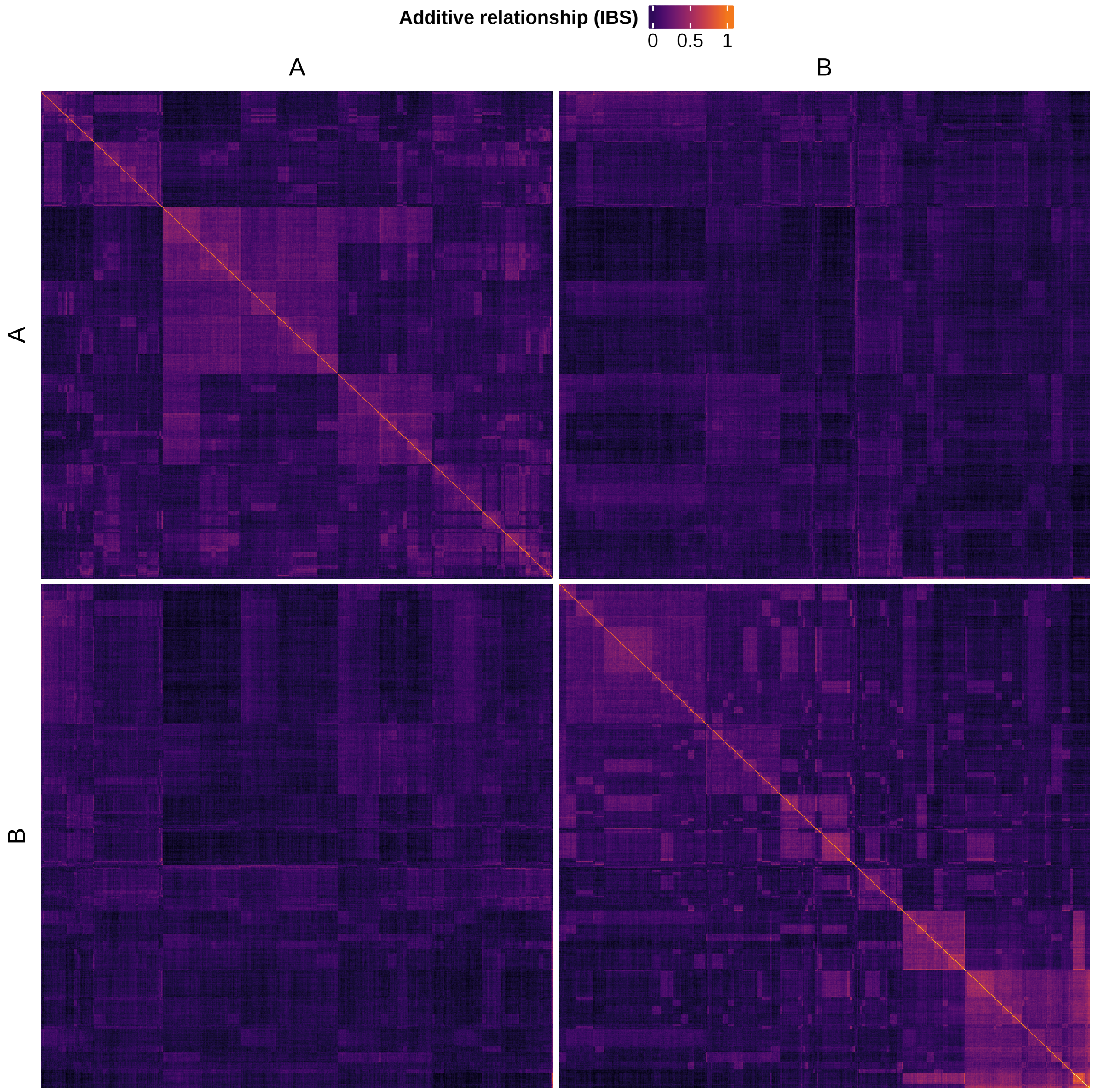

Figure S4: Heatmap representation of the genomic relationship matrix (identity-by-state, IBS), subdivided according to the genetic pools (A and B)

Factor analytic model selection

Table S1: Model selection parameters (Akaike Information Criterion, AIC; and Explained variance, Exp. Var.) of models with different number of factors and strategies (single stage, SS; and two-stage with BLUEs, dBLUPs and dABLUPs as entries, 2S-BLUE, 2S-dBLUP and 2S-dABLUP, respectively). In the the two-stage strategies, diagonal and full weights were used.

Strategy	Weight	Model	AIC	Exp. Var.
SS	-	FA1	40216.84	87.772
		FA2	40211.40	92.694
		FA3	40217.72	95.746
		FA4	40222.78	97.864
2S-BLUE	Diagonal	FA1	31249.73	87.094
		FA2	31248.55	92.834
		FA3	31254.19	94.611
		FA4	31259.60	97.136
	Full	FA1	31271.17	86.659
		FA2	31270.56	93.058
		FA3	31275.78	97.150
		FA4	31281.18	97.758
2S-dBLUP	Diagonal	FA1	30846.62	88.557
		FA2	30848.58	92.057
		FA3	30853.78	94.834
		FA4	30858.61	99.351
	Full	FA1	30726.88	87.356
		FA2	30727.44	91.472
		FA3	30730.82	96.452
		FA4	30736.38	98.647
2S-dABLUP	Diagonal	FA1	30840.58	88.354
		FA2	30842.61	91.827
		FA3	30847.72	94.608
		FA4	30852.49	99.085
	Full	FA1	30720.61	87.303
		FA2	30721.14	91.392
		FA3	30724.40	96.304
		FA4	30729.88	98.448

Variance components of the first-stage analysis

In the Tables below, effects that have '-' as components had estimation issues. Estimates of additive genetic variance have been multiplied by a correcting factor (D = 0.97 in the ABLUP and D = 0.99 in the SS) to guarantee comparability.

Table S2: Variance component estimates obtained from the first-stage models without relationship information.

Environment	Effect	Estimates
OT1NAM22	Genotypic	32.114
	Row.group	0.590
	Col.group	4.333
	Intersection (block)	0.097
	Error	87.226
	Autocorr (row)	0.222
	Autocorr (col)	-
OT1RWE21	Genotypic	60.081
	Row.group	0.615
	Col.group	7.843
	Intersection (block)	-
	Error	160.085
	Autocorr (row)	0.067
	Autocorr (col)	0.025
OT1RWE22	Genotypic	83.785
	Row.group	-
	Col.group	0.847
	Intersection (block)	-
	Error	193.665
	Autocorr (row)	0.020
	Autocorr (col)	0.042
OT1SER22	Genotypic	32.452
	Row.group	1.409
	Col.group	7.149
	Intersection (block)	-
	Error	52.750
	Autocorr (row)	0.297
	Autocorr (col)	-
	Genotypic	26.634
	Row.group	-
	Col.group	0.067

(continued)

Environment	Effect	Estimates
OT2NAM21	Intersection (block)	-
	Error	15.655
	Autocorr (row)	0.449
	Autocorr (col)	-
OT2SER21	Genotypic	3.517
	Row.group	0.044
	Col.group	0.410
	Intersection (block)	0.021
	Error	7.374
	Autocorr (row)	0.229
	Autocorr (col)	0.078

Table S3: Variance component estimates obtained from the first-stage models with relationship (pedigree) information.

Environment	Effect	Estimates
OT1NAM22	Additive	18.549
	Non-additive	15.355
	Row.group	0.660
	Col.group	4.661
	Intersection (block)	0.191
	Error	84.876
	Autocorr (row)	0.214
	Autocorr (col)	-
OT1RWE21	Additive	24.019
	Non-additive	42.781
	Row.group	0.402
	Col.group	8.167
	Intersection (block)	-
	Error	154.724
	Autocorr (row)	0.084
	Autocorr (col)	0.022
OT1RWE22	Additive	16.418
	Non-additive	67.983
	Row.group	-
	Col.group	0.764
	Intersection (block)	-
	Error	193.344
	Autocorr (row)	0.017
	Autocorr (col)	0.044
OT1SER22	Additive	3.392
	Non-additive	29.235
	Row.group	1.432
	Col.group	7.199
	Intersection (block)	-
	Error	52.704
	Autocorr (row)	0.300
	Autocorr (col)	-
	Additive	9.684
	Non-additive	17.679
	Row.group	-

(continued)

Environment	Effect	Estimates
OT2NAM21	Col.group	0.040
	Intersection (block)	-
	Error	15.345
	Autocorr (row)	0.442
	Autocorr (col)	0.015
OT2SER21	Additive	0.714
	Non-additive	2.864
	Row.group	0.043
	Col.group	0.423
	Intersection (block)	0.041
	Error	7.343
	Autocorr (row)	0.239
	Autocorr (col)	0.073

Table S4: Variance component estimates obtained from the single-stage model.

Environment	Effect	Estimates
OT1NAM22	Additive	16.827
	Non-additive	20.916
	Row.group	0.771
	Col.group	5.433
	Intersection (block)	0.243
	Error	80.545
	Autocorr (row)	0.272
	Autocorr (col)	-
OT1RWE21	Additive	40.523
	Non-additive	47.699
	Row.group	0.255
	Col.group	8.028
	Intersection (block)	-
	Error	135.851
	Autocorr (row)	0.135
	Autocorr (col)	0.036
OT1RWE22	Additive	36.358
	Non-additive	60.120
	Row.group	-
	Col.group	0.618
	Intersection (block)	0.167
	Error	184.825
	Autocorr (row)	0.050
	Autocorr (col)	0.030
OT1SER22	Additive	2.638
	Non-additive	29.690
	Row.group	1.192
	Col.group	7.581
	Intersection (block)	-
	Error	52.254
	Autocorr (row)	0.293
	Autocorr (col)	-
	Additive	10.422
	Non-additive	17.390
	Row.group	-
	Col.group	0.106

(continued)

Environment	Effect	Estimates
OT2NAM21	Intersection (block)	-
	Error	14.951
	Autocorr (row)	0.433
	Autocorr (col)	0.026
OT2SER21	Additive	0.925
	Non-additive	2.558
	Row.group	0.003
	Col.group	0.467
	Intersection (block)	0.102
	Error	7.574
	Autocorr (row)	0.239
	Autocorr (col)	0.054

Model comparison: total genotypic value

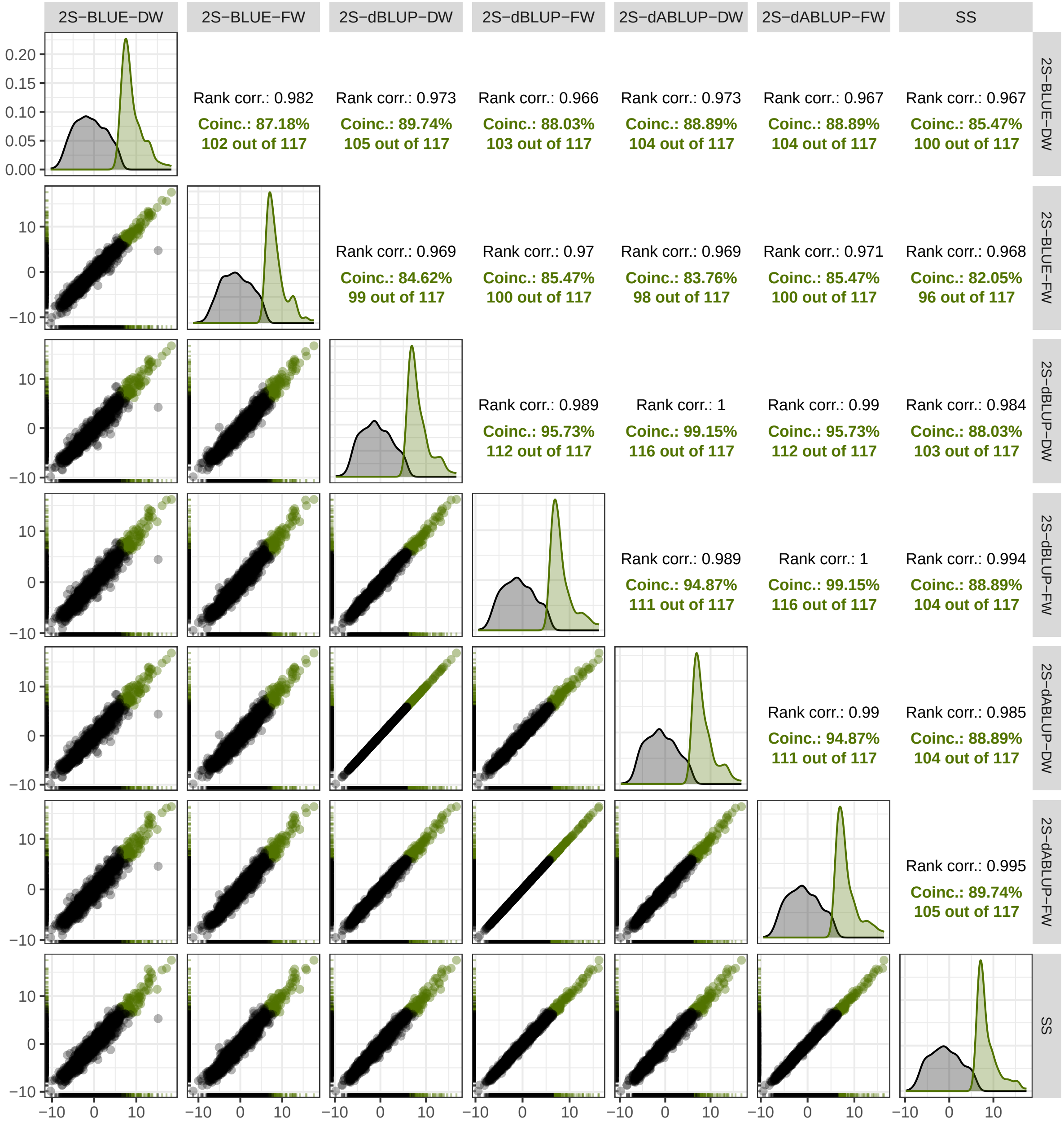

Figure S5: Relationship between total genotypic values (TGVs) obtained from two- (prefix "2S") and single-stage ("SS") models. The column (top) and row (right) titles represent the adopted strategy. In two-stage strategies, "BLUE", "dBLUP" and "dABLUP" are the entries used in the second-stage model. "DW" and "FW" distinguish if a diagonal or a full weight matrix was used. The plots in the diagonal contain the distribution of all GEBVs (in black), and of the top 10% candidates (in green). The scatter plots in the lower triangle have the relationship between GEBVs of different models. Each grey dot represents a candidate's GEBV, and the green dots are the candidates that were amongst the top 10% according to both strategies. This relationship is quantified in the upper triangle, which contains the ranking correlation ("Rank corr.") between GEBVs (in black), and the coincidence between the top 10% candidates of both strategies.
